# FASTory assembly line power consumption data

**DOI:** 10.1016/j.dib.2023.109160

**Published:** 2023-04-19

**Authors:** Mahboob Elahi, Samuel Olaiya Afolaranmi, Wael M. Mohammed, Jose Luis Martinez Lastra

**Affiliations:** FAST-Lab, Faculty of Engineering and Natural Sciences, Tampere University, Finland

**Keywords:** Power consumption-based prognostics model, Predictive maintenance, Power consumption, Conveyor belt deterioration, Belt tension, Discrete manufacturing systems

## Abstract

Machine learning (ML) techniques are widely adopted in manufacturing systems for discovering valuable patterns in shopfloor data. In this regard, machine learning models learn patterns in data to optimize process parameters, forecast equipment deterioration, and plan maintenance strategies among other uses. Thus, this article presents the dataset collected from an assembly line known as the FASTory assembly line. This data contains more than 4,000 data samples of conveyor belt motor driver's power consumption. The FASTory assembly line is equipped with web-based industrial controllers and smart 3-phase energy monitoring modules as an expansion to these controllers. For data collection, an application was developed in a timely manner. The application receives a new data sample as JavaScript Object Notation (JSON) every second. Afterwards, the application extracts the energy data for the relevant phase and persists it in a MySQL database for the purpose of processing at a later stage. This data is collected for two separate cases: static case and dynamic case. In the static case, the power consumption data is collected under different loads and belt tension values. This data is used by a prognostic model (Artificial Neural Network (ANN)) to learn the conveyor belt motor driver's power consumption pattern under different belt tension values and load conditions. The data collected during the dynamic case is used to investigate how the belt tension affects the movement of the pallet between conveyor zones. The knowledge obtained from the power consumption data of the conveyor belt motor driver is used to forecast the gradual behavioural deterioration of the conveyor belts used for the transportation of pallets between processing workstations of discrete manufacturing systems.

Specifications table

**Table:** 

Subject	Industrial Engineering, Manufacturing

Specific subject area	Energy data which includes the power consumption of a conveyor belt motor driver
Type of data	CSV, JSON, Table, Figure
How the data were acquired	The data is collected from the FASTory assembly line, which is equipped with web-based Remote Terminal Units (RTUs) and smart 3-phase energy monitoring modules as an expansion to the RTUs. A software application was developed, which invokes REST energy services and subscribes to REST-formatted HTTP event notifications for smart 3-phase energy monitoring module. After every second, the application receives a new data sample as JavaScript Object Notation (JSON) and extracts the energy data for relevant phase. The extracted data is stored in a MySQL database for the purpose of processing at a later stage [1,2].
Data format	Raw, Unprocessed and Processed
Description of data collection	The collected data is intended for the development of a machine learning (ML) model, which utilizes the power consumption of the conveyor belt motor driver and the load on the conveyor to predict a belt tension class. This model learns the power consumption pattern of the conveyor belt motor driver under different belt tension values and load conditions on the conveyor (i.e., number of pallets). During data collection, the tension of the conveyor belt and the load were varied from zero to a maximum value [2]. It involves the following: The conveyor belt driver power consumption records were retrieved from the MySQL database and saved as comma separated values in a csv file. This is an unprocessed data with the following fields: RMS Current, RMS Voltage, Load Combinations, %Belt Tension, Power (W), and %Power/Nominal_Power. The unprocessed data was processed with Python data processing libraries like “pandas” and “scikit-learn”. The processed data was then saved in a new csv file. The processed data has all fields similar to the unprocessed data except the following fields: Load, Class_3, Normalized_Power, and Normalized_Load. 500 test samples were extracted randomly from the processed data and exposed to the ML model. Afterwards, the model was trained, tested, and validated using the remaining data samples. Separate Python jupyter notebooks were created for data visualization, data processing, and training the belt tension predictor ANN model [3].
Data source location	FASTory Assembly Line Future Automation Systems and Technologies Laboratory (FAST-Lab) Tampere University Tampere, Finland
Data accessibility	Dataset:https://doi.org/10.23729/24df28ed-f7b4-482d-9458-b708485e7cb8[2] Software (jupyter notebooks for data visualization, processing, and model training): https://zenodo.org/badge/latestdoi/492717954[3]
Related research article	Elahi, Mahboob, Samuel Olaiya Afolaranmi, Wael M. Mohammed, and Jose Luis Martinez Lastra. "Energy-Based Prognostics for Gradual Loss of Conveyor Belt Tension in Discrete Manufacturing Systems." *Energies* 15, no. 13 (2022): 4705. doi: https://doi.org/10.3390/en15134705[6]

## Value of the Data

• The collected data includes more than 4,000 power consumption data samples of conveyor belt motor driver of the FASTory assembly line. This data can be used to build an AI-powered model, which monitors the gradual behavioural deterioration of the equipment, belt wear and tear, and gradual loss of belt tension of conveyor belts in discrete manufacturing system. At the time of conducting this research, power consumption data related to the prediction of the gradual loss of belt tension was not available publicly. Hence, the need to conduct the research and gather the data samples.
• The data can be utilized by researchers and data engineers for comparing equipment/sensor model-driven and data-driven models to shift plant maintenance strategy from Run-2-Failure and Preventive to Predictive maintenance strategy. In essence, for researchers aiming to predict the gradual loss of belt tension through mathematical modelling-based approach, the approach utilized in this research offers a basis for comparison.
• The data can be used with the digital twin of a conveyor belt operated transportation system to get insight into the effect of insufficient, sufficient, and abundant belt tension on the required necessary traction force to overcome conveyance path friction, material transfer time between workstations, belt wear and tear, material slippage, and stress on the motor driver shaft, etc. Furthermore, the data can be used to develop a data-driven prognostic model to predict the remaining useful life of equipment. In the case of this research, the data has been used to develop a model for the prediction of the belt tension class relating to “low”, “optimal” and “over tense” classes. The predictions could be regularly stored in a database and analyzed to estimate the operating time of the belt for each belt tension class. If the belt operated time for the “low” and “over tense” classes is above the specified threshold value, the belt may be at the end of its useful life.

## 1 Objective

The data presented in this article has been collected for the purpose of building a prognostic model for monitoring the continuous degeneration of conveyor belts in a discrete manufacturing system based on power utilization patterns. The data was collected from an assembly line that is equipped with smart energy meters, under two different cases, namely static and dynamic cases. In the static case, the data gathered was used to build a data-driven model capable of learning the power consumption pattern of the conveyor belt motor driver under varying belt tensions and load conditions, while in the dynamic case, the relationship between the belt tension and the pallet transportation was examined. This data article adds value to the original research article [6] by making data available for studying, understanding, and establishing the relationship between the health of a conveyor belt and its remaining useful life, which is particularly important for the predictive maintenance of the conveyor belt. Specifically, the data may be utilized for the development of data-driven models for predicting the remaining useful life of conveyor belts.

## 2 Data Description

In Table 1, the data files and python jupyter notebooks used for data pre-processing and training the ANN model are presented. The description of each data file and python jupyter notebooks are also provided in Table 1.

**Table 1:** Data and jupyter notebook files.

File name	Description
FASToryPowerConsumptionData_UnProcessed.csv	Datafile containing the data samples before data pre-processing
FASToryPowerConsumptionDataVisulation.ipynb	Python code used for data plotting
FASToryPowerConsumptionDataProcess.ipynb	Python code for data pre-processing
FASToryPowerConsumptionData_Processed.csv	Datafile containing the data samples after data pre-processing
FASToryPowerConsumptionData_TraningData.csv	Datafile used for ANN training
FASToryPowerConsumptionData_TestData.csv FASToryANNbeltTensionPredictorModel.ipynb	Unseen samples used for testing ANN model Python code used for training the ANN model

**Fig. 1:**
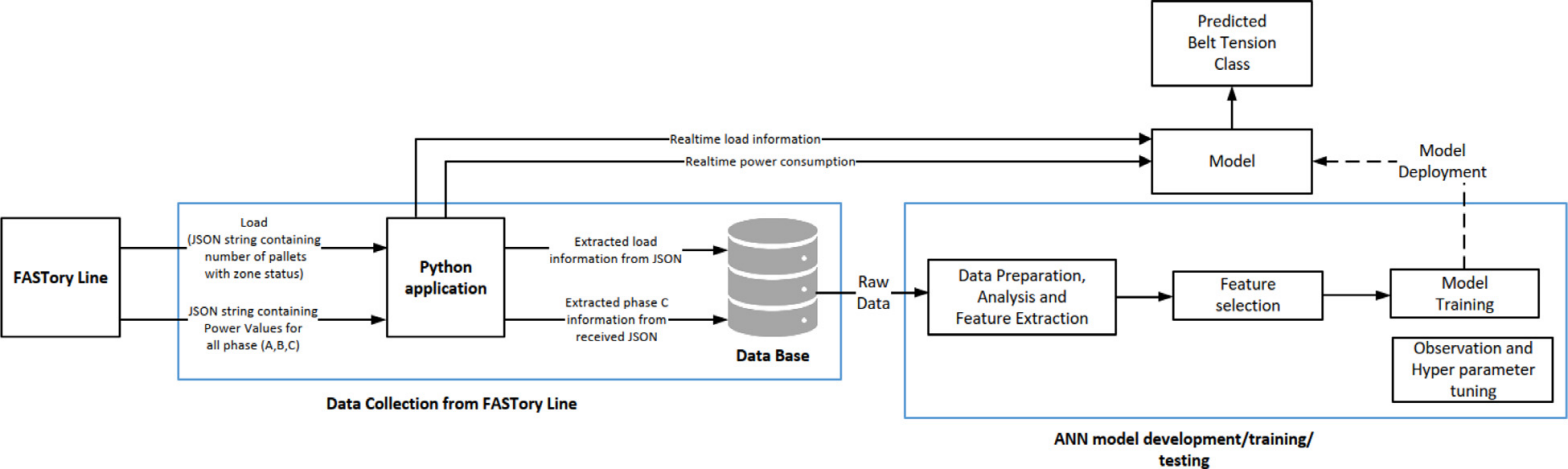
Main steps for development of the data-driven prognostic model [6].

Fig. 1 shows the main steps involved in the collection of data and the development of the ANN model, that predicts the belt tension class. Firstly, a python application was developed to communicate with the FASTory assembly line for the purpose of data collection in JSON format. The collected data samples provide information about load and power consumption for Phases A (Robot controller), B (Cabinet I/O) and C (Conveyor belt motor driver). The python application extracts load and power consumption information for Phase C from incoming samples and stores it in a MySQL database. Once the ANN model is deployed, the application provides real-time samples as input to the ANN model to get real-time prediction on belt tension class.

**Fig. 2:**
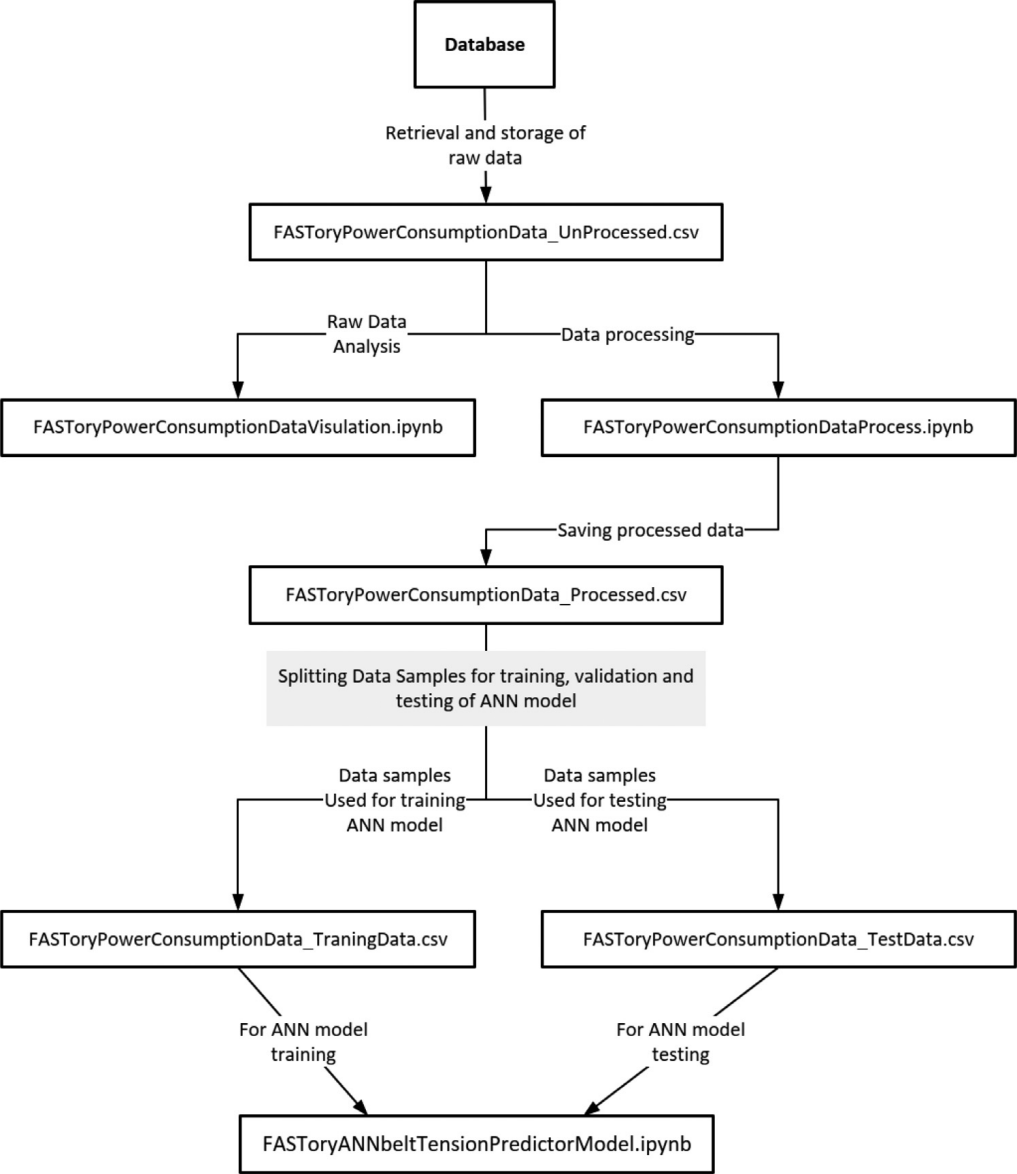
Datafiles for pre-processing and ANN model training.

In Fig. 2, the procedures involved in the generation and utilization of the datafiles is presented. The process begins with the extraction of the raw power consumption data from the database and ends with the development of the belt tension predictor model. The development of the ANN model involves the use of the python jupyter notebook (an interactive development environment for notebooks, code, and data) as all data preprocessing, visualization, and model training were done with jupyter notebook files. The raw power consumption data samples retrieved from the database are stored in csv format in the file named *“FASToryPowerConsumptionData_UnProcessed.csv”*. This allows for the utilization of the data samples at a later stage for data visualization and data processing. The *“FASToryPowerConsumptionDataVisulation.ipynb”* python jupyter notebook file contains the code used for plotting the power consumption data for visualization (i.e., visualization of the power consumption patterns of the conveyor belt motor driver). The Python jupyter notebook file named *“FASToryPowerConsumptionDataProcess.ipynb”* contains the code for retrieving the power consumption data from the database, pre-processing (data transformation, feature scaling, data labeling etc.) the data and splitting the whole data into training and testing samples for the purpose of model training and testing. Following the processing of the raw data samples, the processed data samples were stored in a new file named *“FASToryPowerConsumptionData_Processed.csv”*. This processed data samples were divided into two separate files for training, validation, and hyperparameter tuning (*“FASToryPowerConsumptionData_TraningData.csv”*) and testing (*“FASToryPowerConsumptionData_TestData.csv”*). To train the ANN model, data samples from the *“FASToryPowerConsumptionData_TraningData.csv”* file were used. After the training, the model accuracy was checked by using data samples from the *“FASToryPowerConsumptionData_TestData.csv”* file. The *“FASToryANNbeltTensionPredictorModel.ipynb”* contains the code for training and testing the ANN model.

### 2.1 Experimental Design, Materials, and Methods

**Fig. 3:**
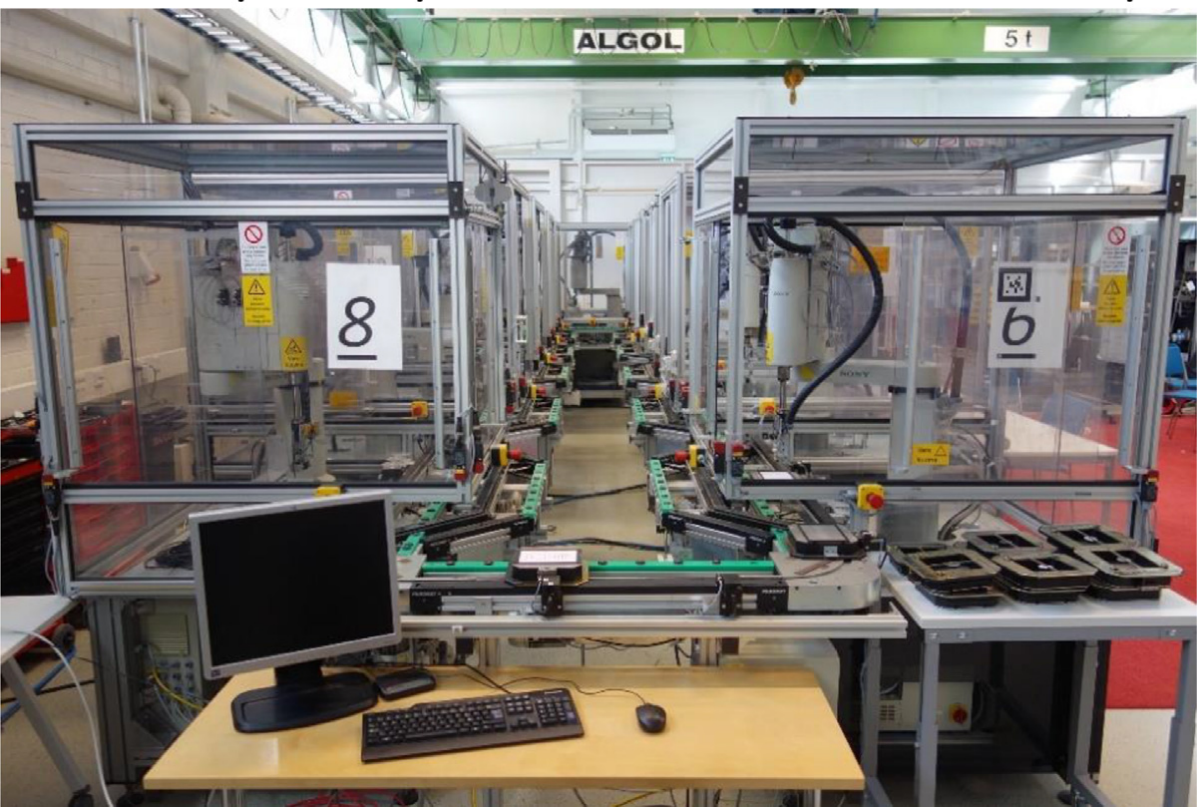
FASTory assembly line.

In this research work, data is collected from the FASTory assembly line shown in Fig. 3 [4], and which is located at FAST-Lab Tampere University, Finland. In late 90s, the FASTory assembly line was used for assembling different Nokia cell phone models. Later, the assembly line was relocated to Tampere University and retrofitted with smart web-based remote terminal units (RTUs) known as S1000, and E10 smart 3-phase energy monitoring modules as an expansion unit [1]. The FASTory assembly line now imitates phone assembly operation by drawing different phone models. It has 10 identical workstations, with a robot in each that draws the main components (frame, screen, and keyboard with different colours and shapes) of three models of a mobile phone on a sheet of paper carried by a pallet. This combination results in the production of 729 variants (i.e., 9 different screens * 9 different keyboards * 9 different frames). The pallet is carried by a conveyor, whose track selector is actuated by air pressure.

**Fig. 4:**
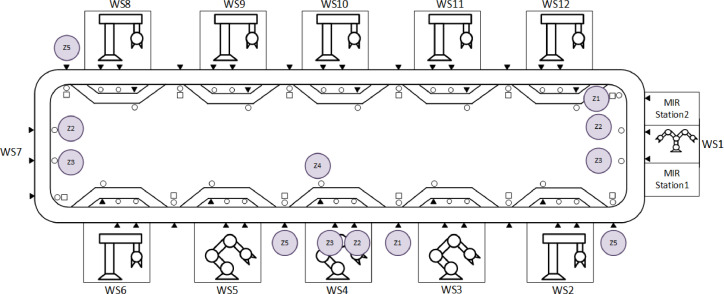
FASTory line layout.

Fig. 4 presents the layout of the FASTory assembly line. The FASTory assembly line workstations (numbered from 2 to 6 and 8 to 12) are used for executing the drawing operations, one workstation (numbered as 1) for loading and unloading the papers and one workstation (numbered as 7) for loading and unloading pallets. Each workstation is equipped with RFID for recognizing the incoming pallets. In addition, each workstation (2–6 and 8–12) includes a main conveyor and a bypass conveyor. The two conveyors split into different zones which are marked in Fig. 4 as 1,2,3,4,5 and referred to as Z# in this article. The entry and exit points of the workstations are located at Z1 and Z5 respectively. The main conveyor has 4 zones, (Z1, Z2, Z3, Z5), and for each zone, there is a presence sensor for checking the presence of a pallet and a stopper for stopping the pallet when it arrives. Z3 is the production zone of each workstation. The Z1 of each workstation has a RFID tag reader which is used to read the pallet ID. The Z1 of the current workstation and Z5 of the next workstation are the same. The bypass conveyor has one zone (Z4) and one stopper.

**Fig. 5:**
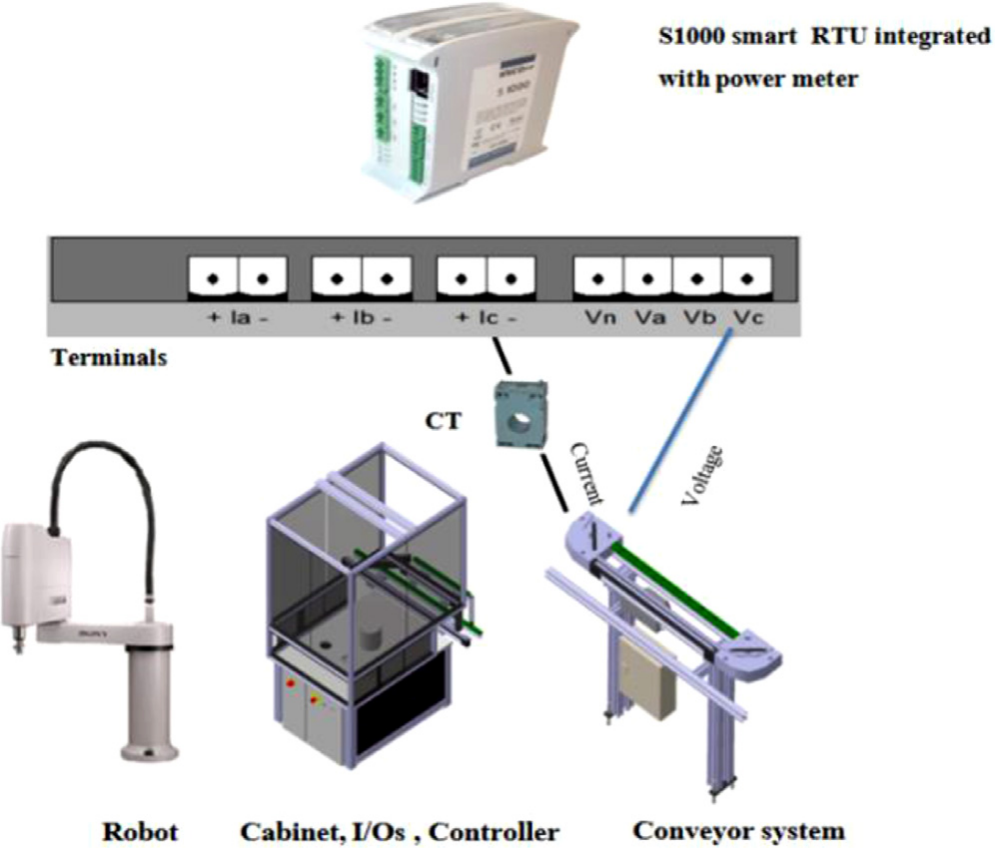
S1000-E10 module for collecting power measurements [5].

The E10 smart 3-phase energy monitoring modules is shown in Fig. 5. It monitors the energy levels by measuring the energy consumption of the 3-phase AC motor. Phase A is assigned to the robot, Phase B is allocated to the cabinet, I/Os, and the controller, while Phase C is assigned to the conveyor system (including main and bypass). Power is measured by sampling the current and voltage. The current is sampled by a current transformer (CT) connected to +Ia-, +Ib- and +Ic- terminals and the voltage is measured by direct connection of the 3 phases (Va, Vb and Vc) and the neutral (Vn) terminals of the E10 expansion module [5]. The Phase C energy values are of interest in this article (see Fig. 5).

For data collection, a software application was developed, which invokes REST energy services and subscribes to REST-formatted HTTP events. After every second, the application receives a new data sample as JavaScript Object Notation (JSON), extracts energy data for the relevant phase (i.e., Phase C) and inserts the extracted data fields in a MySQL database for the purpose of processing at a later stage [1,2]. Table 2 provides information about the events exposed by the RTUs. These event notifications provide information about the energy consumption (via S1000 energy meters) and CAMX state events (e.g., pallet input to a conveyor piece etc.).

**Table 2:** Event notification from S1000-E10 module [6].

S/N	Event Notification	Description
1	EnergyMeter	The energy consumption of the robot/conveyor/controller in each working cell. It is published at a time interval of one second
2	DrawStart/DrawEnd	This includes information about the Cell ID (i.e., workstation number), recipe number, pen colour, and time stamp
3	EquipmentState	This includes information about the Cell ID, state of conveyor zones (“0” or “1”), pallet ID, and time stamp

**Fig. 6:**
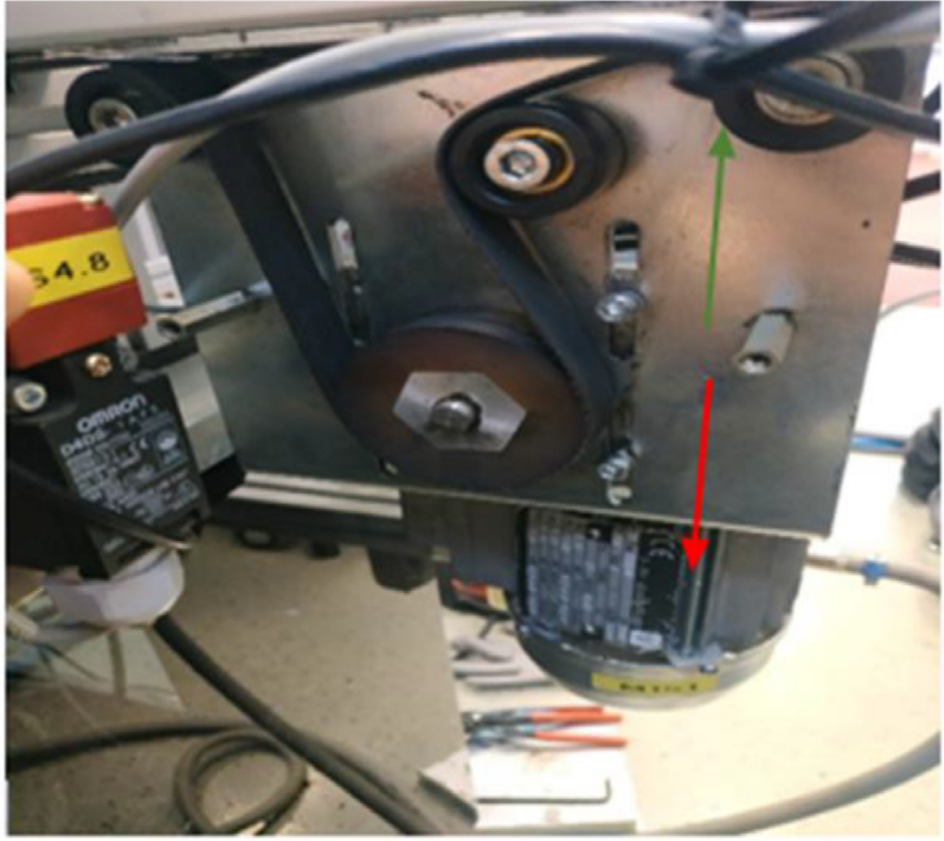
Motor driver with head pulley at main conveyor [6].

During data collection, the tension of the conveyor belt and the load on the conveyor were varied according to Table 3 and Table 4. Table 3 shows the values of the head pulley position and the corresponding percentage belt tension as the tension in the conveyor belt was varied from zero to a maximum value by changing head pulley position from the reference point. The head pulley can move from 0 to 2.7cm. In Fig. 6, a visual illustration of the range of distance, with which the head pulley can be repositioned is provided. The tension in the belt decreases in upward direction and increases in downward direction. Table 4 provides information about the pallet positions and the active zones on the main conveyor based on different load combinations. The “1” and “0” in the “Active zone” column in Table 4 respectively indicates the presence and absence of a pallet on a zone on the main conveyor.

**Table 3:** Head pulley position and % belt tension [6].

Head pulley position (cm) from initial point	%Belt tension
0	0
0.4	15
0.81	30
1.22	45
1.62	60
1.89	70
2.02	75
2.29	85
2.43	90
2.57	95
2.7	100

**Table 4:** Pallet position on main conveyor's zones with respect to load combinations [6].

Load Combination	Active Zones	Description
0	0000	No load
1	1000	1 pallet at Z1
2	0100	1 pallet at Z2
3	1100	2 pallets; 1 pallet at each zone (Z1, Z2)
4	0010	1 pallet at Z3
5	1010	2 pallets; 1 pallet at each zone (Z1, Z3)
6	0110	2 pallets; 1 pallet at each zone (Z1, Z3)
7	1110	3 pallets; 1 pallet at each zone (Z1, Z2, Z3)
8	0001	1 pallet at Z5
9	1001	2 pallets; 1 pallet at each zone (Z1, Z5)
10	0101	2 pallets; 1 pallet at each zone (Z1, Z5)
11	1101	3 pallets; 1 pallet at each zone (Z1, Z2, Z5)
12	0011	2 pallets; 1 pallet at each zone (Z3, Z5)
13	1011	3 pallets; 1 pallet at each zone (Z1, Z3, Z5)
14	0111	3 pallets; 1 pallet at each zone (Z2, Z3, Z5)
15	1111	4 pallets; 1 pallet at each zone (Z1, Z2, Z3, Z5)

For this research work, data was collected for two different cases i.e., static, and dynamic cases.

1. In the static case, the conveyor belt runs continuously irrespective of whether the pallets residing on conveyor belt were stopped via stoppers or not, and which increases the friction along the conveyance path. This data is used by the ML model for learning the power consumption pattern of the conveyor belt motor driver for different belt tensions and load conditions.
2. In the dynamic case, the belt tension was kept constant, and the pallets were moved between zones to investigate how the belt tension affects the movement of pallets between conveyor zones as well as the transportation of material/tool/equipment between workstations. This case is further divided into two cases: In the first case, a pallet moves from source to destination zones on the main conveyor with no other pallet residing on the remaining zones. In the second case, one or more pallets resides on the conveyor zone(s) when a pallet moves from the source zone to the destination zone.
3. The description of the fields of the static case data (i.e., unprocessed, and processed data) and the dynamic case data are provided in Table 5. The processed data has three new fields while the remaining fields are same as the unprocessed data.

**Table 5:** Description of fields of static and dynamic case data.

Field	Data type	Description
Unprocessed Static Case Data		
RMS Current (A)	Float	Rms Current
RMS Voltage (V)	Float	Rms Voltage
Load Combinations	Int	A number between 0 and 15, which when converted to binary gives the zone numbers where the pallet is residing (Active Zones)
%Belt Tension	Int	A number between 0 and 95, representing the belt tension
Power (W)	Float	Power consumed by conveyor belt motor driver
%Power/Nominal_Power	Int	$\text{Nominal}\;\text{power}=\frac{\text{Power}}{\text{rated}\;\text{power}}*100$
Load	Int	The number of pallets per time on the conveyor belt. It is a number between 0 and 4

Processed Static Case Data		

Class_3	Int	Represents the belt tension class label. A number between 1 and 3
Normalized_Power	Float	Normalized power values. A value between 0 and 1
Normalized_Load	Float	Normalized load combination values. A value between 0 and 1

Dynamic Case Data		

Belt Tension (%)	Int	A number between 75 and 95, representing the belt tension
Active Zone	String	Indicates the presence of a pallet on a zone of the conveyor belt. The “Active Zone” field can have two values i.e., “No” or “Z#”. “No” means there is no pallet on the conveyor and “Z#” means there is a pallet on a zone on the conveyor. In “Z#”, “Z” stands for zone and “#” is for number. For example, “Z1” means there is a pallet in Zone 1 of the conveyor.
From	Int	A number indicating the source zone
To	Int	A number indicating the destination zone
Avg. Time (s)	Float	Time taken by a pallet to move from the source zone to the destination zone
Distance (m)	Float	Distance between zones of the conveyor.
Speed (m/s)	Float	Conveyor belt speed calculated as: $\frac{\text{Distance}\;\text{between}\;\text{source}\;\text{and}\;\text{destination}\;\text{zone}}{\text{time}\;\text{taken}\;\text{by}\;\text{pallet}\;\text{to}\;\text{move}\;\text{from}\;\text{source}\;\text{and}\;\text{destination}\;\text{zone}}$

## Ethics Statements

This research work adheres to the ethical requirements for publication in Data in Brief. In addition, this research work does not involve studies with animals or humans.

## CRediT authorship contribution statement

**Mahboob Elahi:** Conceptualization, Methodology, Software, Formal analysis, Investigation, Data curation, Writing – original draft. **Samuel Olaiya Afolaranmi:** Formal analysis, Writing – original draft. **Wael M. Mohammed:** Conceptualization, Methodology, Formal analysis. **Jose Luis Martinez Lastra:** Conceptualization, Supervision.

## Declaration of Competing Interest

The authors declare that they have no known competing financial interests or personal relationships that could have appeared to influence the work reported in this article.

## Data Availability

FASToryEnergyConsumptionData-First_release (Original data) (Zenodo) FASTory Energy Consumption Data (Original data) (Etsin)
